# Number perseveration in healthy subjects: Does prolonged stimulus exposure influence performance on a serial addition task?

**DOI:** 10.2478/v10053-008-0127-8

**Published:** 2013-03-15

**Authors:** Vaitsa Giannouli

**Affiliations:** School of Medicine, Aristotle University of Thessaloniki, Greece

**Keywords:** perseveration, serial addition task, color variation, time variation

## Abstract

Perseverative behavior characterizes mainly patients with severe psychopathology, but it can also be observed in healthy individuals. The aim of the reported experiment was to investigate a serial addition task that elicits strong perseverative behavior in normal subjects by examining the significance of perseveration in the final step of this addition task (Gardner, 1971) as a function of time availability. The classical serial addition task, which was used in the experiment, consisted of 4 consecutive digit decreases in the added numbers following a constant digit (1,000 + 40 + 1,000 + 30 + 1,000 + 20 + 1,000 + 10) and required an additive calculation. The main questions were how and if color and time variations could influence perseverative responses in this task and whether memory performance and relevant mathematical knowledge of the participants could have an effect on responses. The sample of subjects participating in the experiment consisted of 300 healthy university students (112 male, 188 female) ranging from 17 to 40 years of age. They were divided in 5 groups of 60 subjects each. A memory digit span and spatial test were administered and relevant scores were taken for each subject of the 5 groups. Obtained results suggest the presence of a strong perseverative error in the final step of the presentation of digits for the large majority of subjects and for all 5 conditions. It seems that time and color changes and the memory span of the participants have no detectable effect on performance on this specific serial addition task.

## INTRODUCTION

*Perseveration* is defined as the uncontrollable repetition of a particular response or as the inability to interrupt a task in progress and shift from one strategy or procedure to another (Pickett, 2000; VandenBos, 2007). Although there is a growing body of research investigating psychopathological persevering behavior (mainly with patients suffering from damage in prefrontal and frontal areas of the brain; see e.g., Bayles, Tomoeda, & Kaszniak, 1985; Neary, Snowden, Northern, & Goulding, 1988; Sandson & Albert, 1987), there has been relatively few research studying perseverative behavior in healthy individuals.

An initial study on this topic was conducted by Brugger and Gardner (1994). The researchers investigated whether young healthy college students gave unjustifiably incorrect responses in a serial addition task that required the addition of eight numbers sequentially (1,000 + 40 + 1,000 + 30 + 1,000 + 20 + 1,000 + 10). This task involving working memory number processing, required a digit change three consecutive times (1,040, 2,070, 3,090), with a final digit change at the end of the series (4,100). The obtained results indicated that a large percentage (70%) of subjects added the numbers repetitively, giving a wrong response (5,000), instead of the correct one (4,100). It seems that perseveration in this task is due to the unjustified repeated application of an addition rule, which ceases to be effective and does not apply for the last step of the addition.

Proios and Brugger (2004) have used the original uncolored (black-and-white) version of the addition task, and a colored version (coloring the thousands) of the same task to a large group (*N* = 222) of English speaking undergraduate students. The procedure was adopted by Brugger and Gardner (1994). On an overhead projector numbers were presented one by one at a rate of 2 s per number. Classes were randomly assigned to the two test versions. The colored version was used as a way to obtain enhanced saliency of the number 1,000 and as a result to reduce the incidence of perseverative responses (errors). Contrary to the researchers’ expectations, the majority of subjects of each of the two groups failed to produce the correct response irrespective of versions. It is of interest that the distinctiveness of the repetitive presentation of the colored number 1,000 seemed to have no positive effect on the response.

Signs of perseverative behavior may be found besides the serial addition perseveration task, for example in two visually administered music tasks (see Giannouli, 2011). In these tasks participants (a group of musicians and a group of non-musicians) were asked to fill in the final meters of a series of changing notes analogous to the numbers of the addition task. The first visually administered music task consisted of seven meters of descending morphologically similar note patterns and demanded the forecast for the eighth meter, while the second task was similar to the first, but demanded the forecast of the sixth meter of a more complex ascending and descending five meter note pattern. The majority of the participants believed that the requested meters in both tasks should be following in the same repetitive ascending and descending manner, regardless of the multitude of possible choices. It seems that perseveration can also be found in healthy individuals participating in experiments involving simple and complex visually administered music notes presented under different instructions. This finding of strong imitative continuation of the way that subjects previously internally responded to the tasks (regardless of their previous music education), gives support in the direction of a generalized inappropriate maintenance of thought in healthy individuals that goes beyond the initial serial addition task (Giannouli, 2011).

The experiment reported in this paper is not a simple replication of previous investigations. It also explores the possible role of potential effects of time and visual (e.g., color) variations of the stimuli. Both seem to be crucial stimulus aspects for attention capturing and working memory involvement (Baddeley, 1999). Yet, they have not been fully investigated for the specific serial addition task referred to in the present paper. It is well known that working memory does not only hold passively received information, but it also processes information for a brief time period. An example of the use of working memory in everyday conditions is mental arithmetic, in which one tries to multiply mentally two 2-digit numbers without using paper and pencil, or a calculator (Gathercole & Alloway, 2008).

It is now established that memory does not only act as a simple, temporary or permanent, storage of information, but also as an active (“working”) process playing an important role on a range of cognitive tasks, such as mental arithmetic, reasoning, problem solving, etc. (Baddeley, 1997). In addition, relevant research shows that the speed with which stimuli are presented plays also an important role on the extent of memory span (Baddeley, 1997, 1999). In this context, the purpose of the present study is to find out whether extending the time of rehearsing information in number adding tasks may improve the performance of the subjects. If it does, this would suggest that the time available for rehearsing could be of importance in a serial addition task. To summarize, the aim of the reported experiment was (a) to investigate whether individual differences of performance in memory span in the auditory and visual modality and in both forward and backward conditions (recall of digits in the same order and vice versa) could have any influence on the performance in the serial addition task and (b) to clarify whether the stimulus duration (presentation time) and color presentation could have an influence on the correctness of the participants’ responses during the classic serial addition task.

## METHOD

A total of 300 undergraduate university students (188 female, 112 male; 140 natural sciences students and 160 humanities students; *M*_age_ = 21.91, *SD* = 4.95), all volunteers, participated in the experiment. All subjects did not report any current or past neurological disorder, and were free from any current or past psychiatric or neurological disorder, head injury or medical condition, which might have caused any cognitive deficiencies and more specifically possible calculation disorders (van Harskamp & Cipolotti, 2003). Subjects were informed, before their participation, that they would see some numbers in the center of a computer screen, which they would have to add one by one without using paper and pencil and without any external aid. Only at the end of the mental calculation they should write down their response about the final sum. They were classified into five groups of 60 subjects each (balanced for age, sex, and educational level) and subjected to five respective conditions. All subjects were tested individually. The presentation of digits involved color changes of the number 1,000 (black for three groups, red for two groups). It also involved presentation time changes in the same number of 1 s (one group), 2 s (two groups), and 15 s (two groups). Here time variations for the presentation of number 1,000 served as a way to find out if enhancing the saliency of the repeated number 1,000, while extending the rehearsal time for the intervening mental calculations (partial sums) could reduce the incidence of perseverative errors. The stimulus numbers and series of presentation (1,000 + 40 + 1,000 + 30 + 1,000 + 20 + 1,000 + 10) were the same for all five conditions, with no time interval between presentation of successive numbers, while color and time for presentation varied as follows:

1. The first group saw each of the eight numbers, which were presented for 2 s in the center of a computer screen, while the number 1,000 was always printed in black.

2. The second group saw the same numbers at the same presentation time, while number 1,000 was printed in bright red in a white background (following Proios & Brugger, 2004).

3. The third group saw each of the numbers at a presentation rate of 5 s per number, while number 1,000 was printed bright red as for the second group.

4. The fourth group saw each of the numbers at a presentation rate of 1 s, while all numbers were colored black as in the classic black-and-white task (Brugger & Gardner, 1994).

5. The fifth group saw each of the numbers at a presentation rate of 15 s, while all numbers were colored black.

All 300 subjects were also tested for auditory and visual-spatial memory span with presenting numbers and Corsi blocks forward and backward in search of finding possible relations between length of digit span and correct responses on the serial addition task. The stimuli used for auditory span were random strings of numbers (in the presented order or forward recall, or in reverse order or backward recall), while for visuospatial memory span use was made of a Corsi-like block-tapping task (Milner, 1971). The digit forward recall involved presentation of spoken sequences of lists of random numbers from 1 to 9 that subjects were asked to recall in the correct serial order. The rate of presentation was one digit per second. The digitbackward recall test was used as a measure of working memory (following Gathercole, Pickering, Ambridge, & Wearing, 2004). In the visual block recall test, participants were asked to look at nine wooden cubes located randomly on a blue board. The test administrator tapped a sequence of the nine blocks and the subject was asked to repeat the same sequence. Testing began with a single block tap and increased by one additional block following the span procedure outlined above. The subjects were first asked to repeat the sequence of taps used by the experimenter. Following this, they were asked to repeat the series of taps in the reverse order. Forward (verbal [digit] and nonverbal [block]) and backward (verbal [digit] and nonverbal [block]) recall tests were used as indicators of participants’ attention and working memory, respectively.

## RESULTS AND DISCUSSION

For the serial addition task, results of this research seem to confirm the findings of previous research by Brugger and Gardner (1994) and those of Proios and Brugger (2004). More specifically, it was found that of the 300 participants, 70 (23.33%) responded correctly (i.e., gave as a response the correct number 4,100), while 230 participants (76.66%) made incorrect responses. We note here that 46 subjects gave a response other than 5,000, but still an incorrect number, while 184 participants gave one incorrect response, that of number 5,000. The predominant incorrect answer was 5,000 (61.3%). It is of interest that the memory span of all participants was found to be “normal” (cf. Table 1).

**Table 1.** Participants’ Memory Span

Memory Span	*M*	*SD*
Visual forward	6.447	1.073
Visual backward	5.660	1.156
Auditory forward	6.407	1.122
Auditory backward	5.573	1.132

The point-biserial correlations between performance (correctness of responses) in the serial addition task and memory span (both visual and auditory, forward and backward) were all non-significant. Thus, correlations between variables were as follows: for visual forward span and correct response (*r* =.047, *p* = .416), for visual backward span and correct response (*r* =.064, *p* = .266), for auditory forward span and correct response (*r* =-.033, *p* = .565), and for auditory backward span and correct response (*r* =.058, *p* = .319). Likewise, point-biserial correlations between perseverative errors in the serial addition task and memory span (both visual and auditory, forward and backward) were all non-significant, too. The correlational coefficients were as follows: for visual forward span and perseverative error (r = -.052, *p* = .371), for visual backward span and perseverative error (*r* =-.001, *p* =.990), for auditory forward span and perseverative error (*r* =-.011, *p* = .852), and for auditory backward span and perseverative error (*r* =-.025, *p* = .666). Although an extensive number of studies emphasized the role of memory on mental calculations of the serial addition type, the present experiment does not seem to provide support for such a view.

The insignificance of memory for serial addition tasks (of the perseverative type) is also indicated by the finding that there were no group differences in digit span performance (forward and backward) between the participants that made a perseverative error and the participants that made no error on this specific serial addition task (cf. Table 2). Statistical analyses did not show any group differences between those who gave the correct answer versus those who committed a perseverative error on visual forward span, *t*(252) = 1.077, *p* = .853; visual backward span, *t*(252) = 1.053, *p* = .912; auditory forward span, *t*(252) = 1.65, *p* = .171; and auditory backward span, *t*(252) = 0.751, *p* =.546.

**Table 2.** Participants Giving Perseverative Errors and Right Responses and Their Memory Span

	Memory span for those who made the perseverative error		Memory span for those who gave the right response	
	*M*	*SD*	*M*	*SD*
Visual forward span	6.57	1.084	6.40	1.082
Visual backward span	5.75	1.22	5.58	1.12
Auditory forward span	6.42	1.24	6.40	1.09
Auditory backward span	5.65	1.19	5.53	1.10

We should add here that no relationship was found between type of presentation and perseverative response, χ^2^(8) = 11.849, *p* = .158. Thus, no differences in correct answers, perseveration errors, visual span forward, visual span backward, auditory span forward, and auditory span backward between the five groups were found. A further finding was that, in line with Proios and Brugger (2004), the color variation (i.e., coloring the repeated number 1,000) did not increase perseverative or nonperseverative errors. In other words, color or duration of presentation had no effect on perseveration error.

This seems to support the existence of a miscalculation process in healthy participants, which interferes with the normal operation of a multidigit calculation process (for percentage of correct and incorrect responses in each condition, see Table 3).

**Table 3.** Percentage of Correct and Incorrect Responses in Each Condition

Conditions	Correct response („4,100”)	Incorrect response („5,000”)	Other incorrect responses
Classic perseveration task (PT = 2 s)	28.333	55	16.666
Colored perseveration task (PT = 2 s)	13.333	70	16.666
Colored perseveration task (PT = 15 s)	20	60	20
(PT = 1 s)	21.666	70	8.333
Perseveration task (PT = 15 s)	33.333	51.666	15

*Note*. PT = presentation time.

A median split statistical analysis of the subjects of the first group (age range 17-28 years) and of the second group (age range 29-40 years) revealed that the older participants were not more prone to committing errors, especially the perseverative error. Also, an independent samples t-test did not reveal any statistically significant difference of correct responses between the two groups. No statistically significant difference was found, also, between younger (*M* = 0.122, *SD* = 0.328) and older adults, *M* = 0.087, *SD* = 0.288; *t*(298) = -0.506, *p* = .293. We should note that grouping the participants according to their fields of study (humanities-social sciences and natural sciences) indicated that there were differences in the correctness of responses in favor of the participants with a good mathematical knowledge (*M* = 1.19, *SD* = 0.396)in comparison with participants having a less advanced mathematical background, *M* = 0.46, *SD* = 0.211; *t*(298) = 3.99, *p* = .000. Overall, the interactions between age groups (younger and older adults), sex groups (male and female), memory span (high and low memory span scorers) and between conditions, were all found to be statistically non-significant. Also, the correlations/group comparisons were not affected by response interval (or color).

## Conclusion

The findings of the experiment described in this paper indicate that an unexpectedly high percentage of healthy individuals commit a specific type of perseverative error in a serial addition task. Possible reasons for the huge percentage of perseverative error responses might be the unjustified application of the addition rule (the subjects’ wrong representation of the final digit change, which they believed that should be made in a repetitive way in the thousands, instead of the hundreds), regardless of color and duration of the number 1,000. It is also clear that no inter-individual differences regarding attention and working memory (as reflected in test scores for forward and backward digit span and Corsi-block test) have any effect on this sort of perseverative behavior. The only variable that might differentiate performance is the knowledge of numbers and binary operations (elementary arithmetic operations of addition, subtraction, multiplication, and division) that we inferred from the basic grouping of our participants according to their different academic disciplines. A total ignorance of numbers provides no explanation of the error because all participants had basic university education and no calculation disorders. However, maybe specific arithmetic memory and attention skills acquired through everyday practice by only those participants with a good mathematical background have the potential to overcome the error. If such findings are further confirmed by more research, they may shed light on working memory processes. On the other hand, the finding that color and time do not affect performance in regard to perseverative errors in our sample, may suggest that in the serial addition task, working memory of healthy subjects is affected more by repetitive position change of numbers than by their general mathematical know-ledge. Subsequent research may reveal similar perseveration errors in other serial multidigit arithmetic calculations or might uncover factors besides number duration and color that influence perseveration errors.

## References

[R1] Baddeley A. (1997). Human memory. Theory and practice..

[R2] Badeley A. (1999). Essentials of human memory..

[R3] Bayles K. A., Tomoeda C. K., Kaszniak A. W. (1985). Verbal perseveration of dementia patients.. Brain and Language.

[R4] Brugger P., Gardner M. (1994). Perseveration in healthy subjects: An impressive classroom
demonstration for educational purposes.. Perceptual and Motor Skills.

[R5] Gardner M. (1971). Mathematical games.. Scientific American.

[R6] Gathercole S. E., Alloway T. P. (2008). Working memory and learning: A practical guide for teachers..

[R7] Gathercole S. E., Pickering S. J., Ambridge B., Wearing H. (2004). The structure of working memory from 4 to 15 years of
age.. Developmental Psychology.

[R8] Giannouli V. (2011). Music in a serial repetition task: Is there perseverative
behavior?. Acta Neuropsychologica.

[R9] Milner B. (1971). Interhemispheric differences in the localization of psychological
processes in man.. British Medical Bulletin.

[R10] Neary D., Snowden J. S., Northern B., Goulding P. J. (1988). Dementia of frontal lobe type.. Journal of Neurology, Neurosurgery, and Psychiatry.

[R11] Pickett J. (Ed.) (2000). The American English heritage dictionary of English language..

[R12] Proios H., Brugger P. (2004). Influence of color on number perseveration in a serial addition
task.. Perceptual and Motor Skills.

[R13] Sandson J., Albert M. L. (1987). Perseveration in behavioral neurology.. Neurology.

[R14] van Harskamp N. J., Cipolotti L., Halligan P. W., Kiscka U., Marshall J. C. (2003). Assessment and treatment of calculation
disorders.. Handbook of clinical neuropsychology.

[R15] VandenBos G. R. (2007). APA dictionary of psychology..

